# Influence of Temperature on the Enantioselectivity of Koga Tetraamines on Amylose Chiral Stationary Phases

**DOI:** 10.3390/molecules19010009

**Published:** 2013-12-19

**Authors:** Hong-Xun Guo, Steven Wu, Jingshun Sun

**Affiliations:** Analytical Research and Development, Amgen, Inc., One Amgen Center Drive, Thousand Oaks, CA 91320, USA; E-Mails: zwu@amgen.com (S.W.); sun_pharm2001@yahoo.com (J.S.)

**Keywords:** Koga bases, enantioseparation, temperature, van’t Hoff plot, thermodynamic, enthalpy-driven, entropy-diven

## Abstract

Enantioseparation is largely based on the formation of transitional complexes, the solvation species, the stationary phase configurations or the diastereomeric complexes formed by analytes and the chiral stationary phase. Temperature and the chemical nature and composition of the eluent play significant roles during that process. In this study; unique temperature-induced behaviors were observed during the enantioseparation of Koga tetraamines, also known as Koga bases, on polysaccharide chiral stationary phases, in which van’t Hoff plots were acquired over a temperature range of 10 °C to 40 °C with 5 °C increments. Koga bases were eluted by a mixture of methanol and 2-propanol with 0.03% triethylamine as a modifier. The van’t Hoff plots are linear in the case of eluent containing equal volumes of methanol and 2-propanol. Increasing 2-propanol concentration from 50% to 85% in volume led to non-linear van’t Hoff plots over the entire temperature range studied. Examination of the individual non-linear plots revealed two linear regions of 10 °C–20 °C and 20 °C–40 °C. Transition from one linear region to the other at 20 °C indicates alterations of chiral stationary phase conformation and/or enantioseparation mechanism as a result of temperature changes.

## 1. Introduction

Chiral separation is one of the most challenging tasks for chromatographers due to the fact that chiral recognition relies on multiple interactions, one of which needs to be stereoselective. Enantiomers will not be separated *i.e.*, or selectively retained, unless the interactions are specific to their chiral characteristics. Temperature plays an important role in enantioselectivity since it affects viscosity, polarity and diffusivity of the mobile phase. Recent studies have indicated that retention factor, k', decreases with increasing temperature [1,2], indicating that lnk' is reversely proportional to absolute temperature (T). Although those studies satisfactorily account for the effect of temperature on the interaction between analyst and chiral stationary phase (CSP), limited information on the actual mechanism of the chiral separation process has been provided, and further work is needed in order to better understand the mechanism of chiral analytes’ selective retention on CSP. Koga bases were chosen for this study due to the fact that they contain two chiral centers that are C_2_-symmetric [3,4]. The thermodynamics and temperature effects on both enantiomeric and diastereomeric selectivity can therefore be evaluated.

Thermodynamic and kinetic properties of interaction of an analyte with a stationary phase can be altered by different eluent composition, which may cause changes in the accessibility and availability of the sorption sites by modifying the shape, size and chemical microenvironment of chiral cavities of CSP. This results in different enantioselectivity and subsequent enantiomeric separation [5]. Wang *et al.* [6] observed structural changes in amylose CSP as a function of mobile phase composition using solid-state NMR. Kasat *et al.* [7] studied the role of solvent in modifying the structure of amylose phase by X-ray diffraction, infrared spectroscopy, and solid-state NMR. 

Thermodynamic correlations are frequently used in the analysis of analogous chemical processes to shed light on certain important features of the underlying physico-chemical phenomena. The distribution coefficient *(K)* of the analyte between the stationary phase and mobile phase in a chromatographic system is a function of the difference in free energy (∆*G*) of the analyte in the two phases, expressed in Equation (1). The vicinity of temperature (T) changes in enthalpy (∆*H*) can be offset by the changes in entropy (∆*S*) so the free-energy change is independent of temperature:

*RT*ln*K* = −∆*G =* − (∆*H* − T∆*S*) (1)

Chromatographic retention is conveniently measured by the retention factor, k', which is related to the thermodynamic equilibrium constant, K, for eluent binding by the equation k' = ΦK, where Φ is the reciprocal phase ratio of the column. Equation (1) can be rearranged so that it gives the van’t Hoff equation:

ln*k*' *=* −∆*G*/RT = −(∆*H/RT*) + (∆*S*/*R*) *+* lnΦ (2)

If the mechanism of the separation process and the corresponding enthalpy remains constant over the investigated temperature range, a plot of lnk' against 1/T, commonly referred to as a van’t Hoff plot, yields a straight line with a slope equal to −(∆*H/R*) and an intercept equal to [(∆*S*/*R*) *+* lnΦ]. The process when analyte molecules transfer from a mobile phase to a stationary phase is enthalpically favorable but entropically unfavorable. In mobile phase, where the molecular interactions are weaker, an analyte has greater freedom. As the analyte is retained by the stationary phase, its entropy is reduced; the entropy change (∆*S*) must be negative. One the other hand, when the analyte molecule is held on a stationary phase by intermolecular forces, energy is released as a result of the interaction, and thus the enthalpy must also be negative; if (∆*H*) is negative, then the first term in Equation (2) will be positive. 

When the mechanism of a separation process undergoes a change mostly caused by a conformation variation of its stationary phase at a certain temperature or temperature range, the enthalpy value will change and affect the enthalpy and entropy terms in Equation (2). The conformation change can be reversible or irreversible, which greatly depends on the characteristics of the CSP and the analytes. The change of conformation may alter the enthalpy or entropy of adsorption, which can result in a non-linear van’t Hoff plot. The process may involve changes of conformation of stationary phase and those of analyte and diastereomeric complexes; changes of multi-type retention mechanisms; and, finally, changes in multiple types of binding sites on CPSs. In view of importance of conformation and solvation in chiral recognition, there may be more non-linear van’t Hoff plots than linear ones. The reason behind observing fewer non-linear van’t Hoff plots lies in the fact that the temperature range within which HPLC operates is relatively narrow. Most van’t Hoff plots are linear over a sufficiently narrow temperature range. 

The effect on selectivity may be predicted by combining the equations for the enantiomer retention factors:

lnα *=* −∆∆*G*/RT = −∆∆*H/RT* + ∆∆*S*/*R* (3)

where ∆∆*H* and ∆∆*S* are the differences in enthalpy and entropy terms for the enantiomers, and α is selectivity or separation factor. 

According to this thermodynamic equation, selectivity is determined by an enthalpic contribution that decreases with temperature and an entropic contribution that is independent of temperature. Based on Equation (3) it is obvious that selectivity decreases as temperature increases. At the isoenantioselective temperature T_isoenantioselective_ the term of −∆∆*H/RT* and ∆∆*S*/*R* cancel each other and lnα becomes zero where there is no enantioselectivity due to anthalpy/entropy compensation [8,9,10]. However, this hypothesis is only valid when conditions such as the structure of transitional complexes, the form of solvation species and the configuration of stationary phases are the same in the studied temperature range and when achiral contributions to retention are absent, as reported by Levkin *et al.* [11]. 

To understand the aforementioned chiral separation phenomena, we have adopted a thermodynamic approach to analyzing chiral retention data obtained from Koga bases [3,4,12] on a polysaccharide phase (the AD-H [amylose tris-(3, 5-dimethylphenylcarbamate)] column) over a temperature range of 10 °C to 40 °C using two different polar organic eluent systems. 

## 2. Results and Discussion

**Figure 1:**
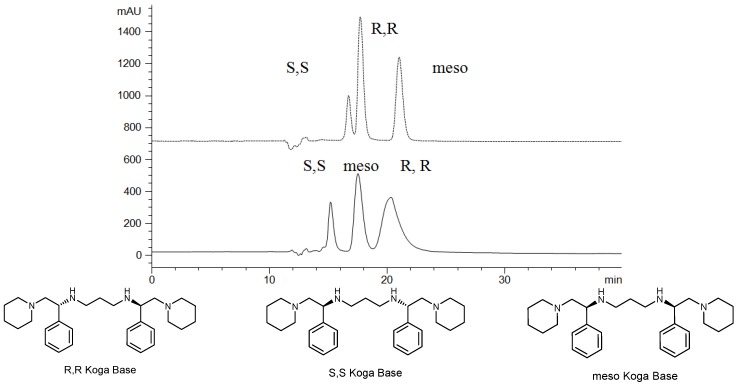
Chromatograms of R, R-; S, S; and meso-Koga base (chemical structure listed below) on a Chiralpak AD-H column, ---- Eluent: 85% -2-propanol-15% methanol-0.03% triethylamine, ----Eluent: 50% -2-propanol-50% methanol-0.03% triethylamine. Flow rate 0.25 mL/min at 20 °C and detection 215 nm [4].

The retention factor and enantiomeric selectivity of Koga bases at different temperatures have been calculated and summarized in Table 1, Table 2, Table 3. The typical enantioseparation of Koga bases and chemical structures of Koga bases are shown in Figure 1 [4].

**Table 1:** Retention factor and enantiomeric selectivity of Koga bases at different temperatures on AD-H column with 50% of 2-propanol and 50% of methanol and 0.03% TEA as eluent.

Temperature (°C)	Koga bases					
	k'			α		
	RR	meso	SS	RR/SS	meso/SS	RR/meso
10	0.88	0.58	0.32	2.76	1.83	1.51
15	0.77	0.52	0.30	2.59	1.74	1.49
20	0.66	0.46	0.27	2.43	1.68	1.47
25	0.61	0.41	0.26	2.37	1.62	1.45
30	0.53	0.37	0.24	2.17	1.52	1.42
35	0.46	0.33	0.23	2.06	1.47	1.40
40	0.41	0.30	0.21	1.96	1.43	1.37

**Table 2:** Retention factor and enantiomeric selectivity of Koga bases at different temperatures on AD-H column with 85% of 2-propanol and 15% of methanol and 0.03% TEA as eluent.

Temperature (°C)	Koga bases					
	k'			α		
	RR	meso	SS	RR/SS	meso/RR	meso/SS
10	0.49	0.83	0.43	1.14	1.71	1.95
15	0.47	0.80	0.41	1.14	1.71	1.96
20	0.46	0.78	0.40	1.15	1.70	1.95
25	0.42	0.69	0.37	1.15	1.64	1.88
30	0.38	0.61	0.34	1.14	1.59	1.81
35	0.36	0.56	0.32	1.14	1.55	1.76
40	0.33	0.49	0.29	1.12	1.52	1.70

**Table 3:** The enthalpy and entropy change as results of enantioseparation of Koga bases.

Eluent	Plot Region **	Koga Base	ΔH (k cal/mol)	Pair of optical Isomer	ΔΔH (k cal/mol)	ΔΔS (kcal/mol. K)
50%IPA–50%MeOH		R, R	−18.230	RR/SS	−8.1	−0.005
		meso	−16.235	RR/meso	−2.3	−0.021
		S, S	−10.163	meso/SS	−5.9	−0.016
85%IPA–15%MeOH	I	meso	−17.062	meso/SS	−5.0	−0.003
		R, R	−12.824	RR/SS	−1.1	−0.009
		S, S	−12.042	meso/RR	−3.9	−0.001
	II	meso	−4.511	meso/SS	−0.1	−0.006
		R, R	−3.879	RR/SS	−1.4	0.001
		S, S	−4.388	meso/RR	−1.5	−0.005

** Region I: Temperature = 20 °C–40 °C; Region II: Temperature = 10 °C–20 °C.

### 2.1. Linear van’t Hoff Plot

The formation of transitional complexes and the solvation species as well as the configuration of the stationary phase or diastereomeric complexes formed by analytes and the CSP depend on the temperature and the solvent system used. In this study, the effect of temperature on retention and resolution of enantiomeric separation of Koga bases was investigated in the temperature range of 10 °C to 40 °C with 5 °C increments. The experiment was performed with two isocratic conditions: 2-propanol–methanol–triethylamine (TEA) 50:50:0.03 (in volume) and isopropanol–methanol–TEA 85:15:0.03 (in volume). The flow rate was 0.25 mL/min in both cases. 

**Figure 2:**
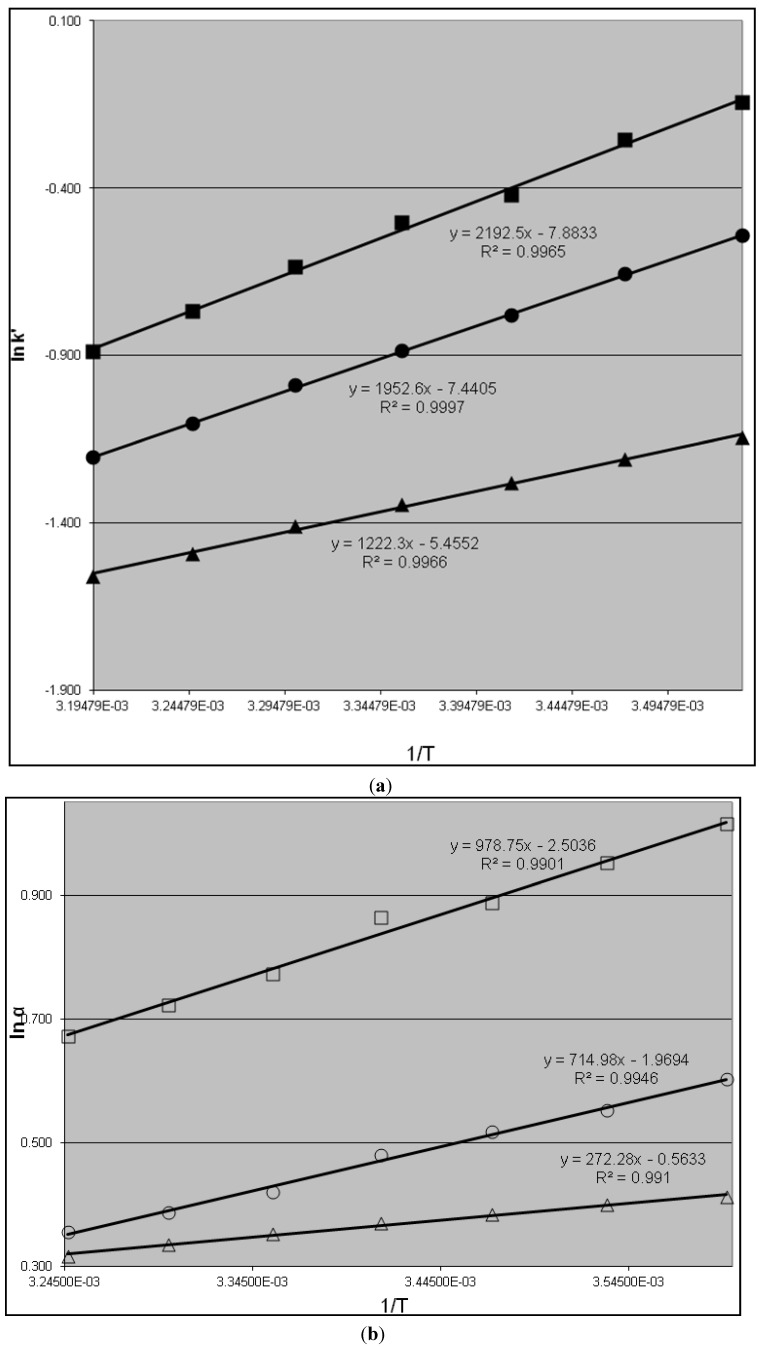
van’t Hoff plots for k' (**a**) and α (**b**) of the *R,R*-, *S,S*- and *meso*-Koga base using equal volume of 2-propanol and methanol, and 0.03% TEA as eluent, flow rate of 0.25 mL/min on a Chiralpak AD-H column (5 um, 250 mm × 4.6 mm). The x-axis is reciprocal of absolute temperature. k' of *R,R*-Koga base, k' of *meso*-Koga base, k' of *S,S*-Koga base; α_RR/SS_ between *R*,*R*- and *S,S*-Koga base, α_RR/meso_ between *R,R*- and *meso*-Koga base, and α_meso/SS_ between *meso*- and *R,R*-Koga base.

Linear van’t Hoff plots involving the retention factor (ln*k*' *versus* 1/T) were observed when the eluent is the mixture of equal volumes of 2-propanol and methanol (Figure 2). The plots of *R,R*-Koga base and meso-Koga base are almost parallel and have positive slopes with negative intercepts, exhibiting minor differences in selectivity as a function of operating temperature [13,14,15]. The plot of *S,S*-Koga base has a smaller positive slope and negative intercept in comparison with *R,R*- or *meso-*Koga base, demonstrating that retention of analytes and enantiomeric and diastereomeric selectivity decrease with increasing temperature. Hence, the enthalpy of association with the CSP is negative and constant over the investigated temperature range for each species of the Koga bases. In addition, the results in Figure 2 demonstrate that the enthalpy remains constant over the studied temperature range, as do physical properties such as viscosity, diffisivity and polarity of the eluent. The chiral separation mechanism essentially remains unchanged over the course of the study. There is no indication of a reversible process that would alter the enthalpy or entropy adsorption under the studied conditions, which is in agreement with the conclusions from previous studies [16,17]. The relative contributions of enthalphy and entropy to the overall free energy remain constant in the process of enantioseparation of Koga bases under the studied conditions.

Enthalpy changes for *R,R*-, *S,S*- and *meso*-Koga base have been calculated and summarized in Table 3. The enthalpy changes of *R,R*-, *S,S*- and *mes*o-Koga base are negative. The differences in enthalpy are important in chiral separation; the bigger the differences, the better the separation from a thermodynamic point of view. The y-intercepts (∆*S*/*R +* lnΦ) are slightly negative in comparison with the enthalpies for the three enantiomers of Koga base. The ∆*S*/*R* is an entropic contribution and the phase ratio, lnΦ, is independent of temperature [18]. Negative intercepts imply that the entropy change of the analyte has resulted in a less random, more orderly state during the process of distribution. Negative values of ∆*H* (as a result of a positive slope) indicate that the retention of Koga bases on CSP is governed by their enthalpic contribution, which decreases with temperature. These values indicate that solute transfer from the mobile phase to the stationary phase is enthalpically favorable but entropically unfavorable. The enthalpy change is related to the strength of interactions between the enantiomers and the mobile phase on one hand and the stationary phase on the other. In the mobile phase, all three species of Koga bases are solvated in an identical way, and hence have equal molar enthalpies. In the stationary phase, enthalpy depends mainly on interactions between specific functional groups and their orientation of the enantiomer and the stationary phase, together with contributions from dispersion and other weak forces. Enantiomeric pairs may have different enthalpies (ΔH) due to the differences in orientation of their functional groups, as well as due to differences in structural compatibility with chiral selective sites. 

The differential changes in molar enthalpy (ΔΔH) and entropy (ΔΔS) in mobile phase are also listed in Table 3. Thus, the enthalpy and entropy changes counteract each other in their impact on analyte retention and enantioselectivity. In most cases, an increase of retention results in enantioseparation. However, a stronger retention may not directly translate to chiral separation if enantioselectivity is not sufficiently high. This is the reason for enantioseparation not necessarily being predominantly “enthalpically driven” or “entropically driven”. In most cases, retention has both enthalpic and entropic components can be carefully adjusted in order to achieve subtle chiral separation. For example, if as a result of its unique configuration one enantiomer can interact more closely with CSP, and in doing so come closer to an energetically interacting group, both the enthalpy and entropy of distribution will change. Consequently, separation of one enantiomer from another will be achieved by both enthapic and entropic contributions to the free energy of distribution, as shown in Equation (3).

### 2.2. Non-Linear van’t Hoff Curves

**Figure 3:**
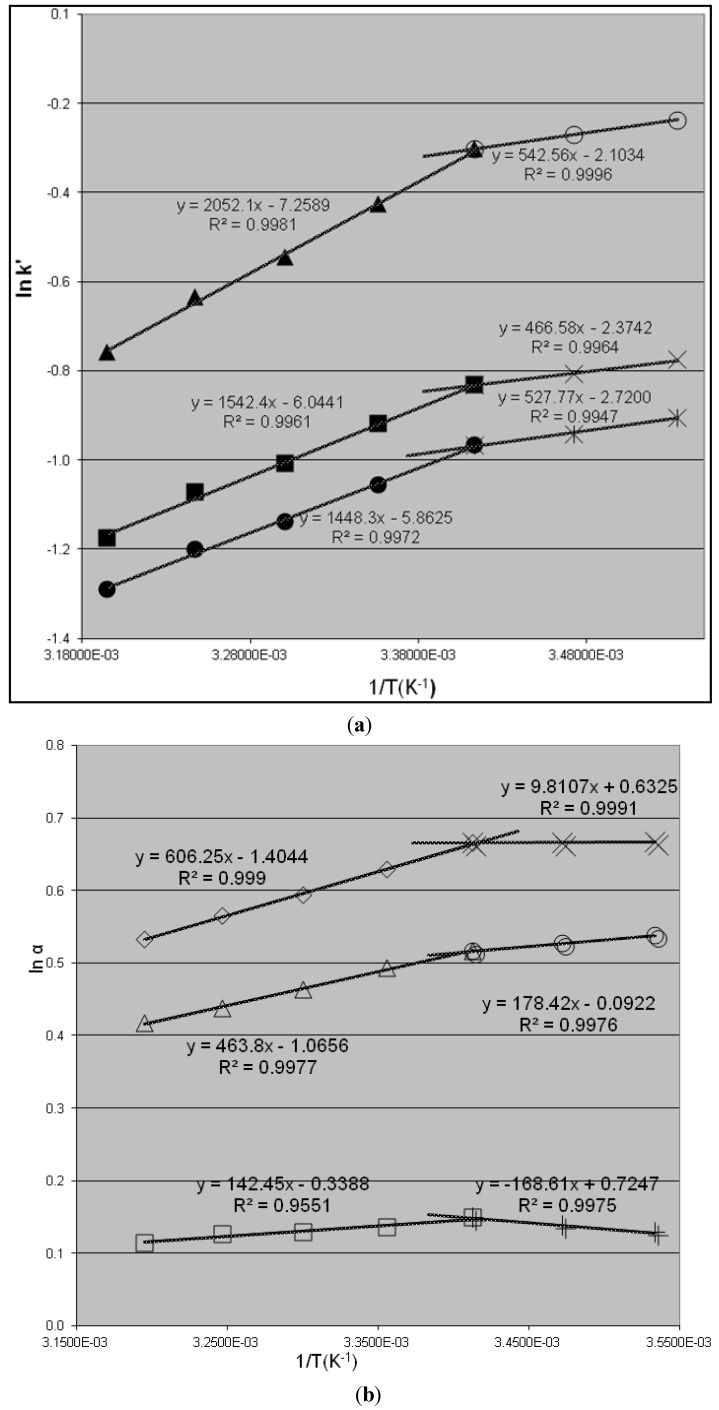
van’t Hoff plots for k' (**a**) and α (**b**) of the *R,R*-, *S,S*- and *meso*-Koga base using 85% 2-propanol-15% methanol, and 0.03% TEA as eluent, flow rate of 0.25 mL/min on a Chiralpak AD-H column (5 um, 250 mm × 4.6 mm). The x-axis is reciprocal of absolute temperature. (**a**) ■ for k' of *R,R*-Koga base (Region I), X for k' of *R,R*-Koga base (Region II); ● for k' of *S,S*-Koga base (Region I), for k' of *S,S*-Koga base (Region II); ▲ for k' of *meso*-Koga base (Region I) and ○ for k' of *meso*-Koga base (Region II); (**b**) □ for α_RR/SS_ between *R,R*- and *S,S*-Koga base (region I), XX α_RR/SS_ between *R,R*- and *S,S*-Koga base (region II); ∆ for α_meso/RR_ between *meso*- and *R,R*-Koga base (Region I), ○ for α_meso/RR_ between *meso*- and *R,R*-Koga base (Region II); and ◊ for α_meso/SS_ between *meso*- and *S,S*-Koga base (Region I), ++ for α_meso/RR_ between *meso*- and *R,R*-Koga base (Region II).

It is interesting to note that van’t Hoff plots (Figure 3) change dramatically when the eluent is changed from a mixture of 50% of methanol and 50% of 2-propanol to a mixture of 15% of methanol and 85% of 2-propanol, with the other chromatographic conditions remaining the same. As shown on Figure 3, the plots of lnk'* versus* 1/T are non-linear for all three species of Koga base in the temperature range of this study. Upon close examination, it is evident that the plots can be divided into two linear regions, which means that van’t Hoff plots are linear in a limited temperature range. The non-linearity of van’t Hoff plot over the entire temperature range can be attributed to changes in the retention mechanism or conformation of the stationary phase as a result of certain reversible process affecting the enthalpy and entropy of adsorption. The process may involve the analyte, the stationary phase, and the mobile phase. Changes in conformation, which alter the way the eluent interacts with either the analyte or CSP, are typically the causes of non-linear van’t Hoff plots. The presence of multiple types of retention mechanisms or bonding sites may also lead to non-linear van’t Hoff plots. The importance of conformation and solvation in chiral recognition and multiple retention processes can be expected [6,16,19,20]. However, this does not lead to non-linear van’t Hoff plots, when such changes are observed; the thermodynamic parameters are no longer independent of temperature. 

In Figure 3, the first region (Region I) occurs between 20 °C and 40 °C, where the plots show a large slope and a small intercept for all three enantiomeric and diastereomeric isomers of the Koga base. The values of the slope and the intercept are of the same order of magnitude as those shown in Figure 2 using a mixture 50% methanol and 50% 2-propanol as eluent. This means that the separation process has similar enthalpy (∆*H*) and entropy (∆*S*) values. Therefore the separation process or mechanism in the Region I is similar to that occuring with a mixture 50% methanol and 50% 2-propanol as eluent. 

The second region (Region II) occurs at the lower temperature range of 10 °C to 20 °C, where the plots have a smaller slope and a smaller intercept for each of the three enantiomeric and diastereomeric isomers of Koga base. This indicates that the separation process has a relatively small ∆∆*H* and a large ∆∆*S* value relative to Region I, which means a larger entropy contribution to the enantioseparation in this region. By comparison, Region II presents a separation process that has a relatively smaller ∆*H* and a large ∆*S* value compared to those obtained in Region I. Enthalpy plays a less significant role than entropy compared to Region I. However, there is not enough evidence to conclude that the chiral separation in Region II is entropy-driven and that the process in Region I is enthalpy-driven [13]. It is clear that a temperature variation can change the process of chiral separation/recognition of the enantiomeric and diastereomeric isomers of Koga base on the same CSP with the same eluent. This type of change suggests that the structure of CSP is temperature dependent; CSP has a different structure in Region I relative to Region II where CSP interacts with Koga base molecules through a different mechanism. There is no (or very little) solvation effect since Region I and II are determined by temperature ranges. 

Thermodynamic parameters obtained from slopes of van’t Hoff plots for Koga base optical isomers are summarized in Table 3. Negative changes of enthalpy for all three enantiomeric and diastereomeric isomers are greater when eluent consists of equal volumes of 2-propanol and methanol than when the eluent composition is 85% 2-propanol-15% methanol. This correlates well with the proton donating ability of the eluent since methanol has a stronger ability for H-bonding than 2-propanol. In terms of interaction with stationary phase, the order from strong to weak is *R,R*-, *meso*- and *S,S*-Koga base, as evidenced by magnitude of negative change in molar enthalpy observed for both eluent systems. This leads to the conclusion that enantioseparation results from formation of transitional complexes and solvation species as well as from configuration of stationary phases or diastereomeric complexes between analytes and CSP, all of which depend on the temperature and solvent system used as eluent.

## 3. Experimental

### 3.1. Equipment

The experiment was carried out on an Agilent 1100 HPLC system (Agilent, Waldbronn, Germany). The column used was a Chiralpak AD-H [amylose tris-(3,5-dimethyl-phenylcarbamate)] 250 mm × 4.6 mm, 5 µm from Chiral Technologies Inc., (West Chester, PA, USA).

### 3.2. Reagents

Methanol (MeOH) and 2-propanol were obtained from Burdick & Jackson (Muskegon, MI, USA), and triethylamine were obtained from J. T. Baker (Central valley, PA, USA). The reagents were ACS grade or better.

### 3.3. Sample Preparation

*S,S*-; *meso*- and R,R-Koga base were synthesized in house. Eluents were used to dissolve the sample at an appropriate concentration.

### 3.4. Mobile Phase Preparation

All mobile phases consisted of two solvents prepared in separate bottles. All the individual solvent bottles contained the designated solvent and 0.03% TEA (*v/v*). The solvent was mixed on-line at the designated ratio, as specified in the text. 

### 3.5. Chromatography

The flow rate was 0.25 mL/min. The retention factor, *k*', and separation factor, α, for each stereoisomer were calculated.

## 4. Conclusions

Van’t Hoff plots of lnk' and lnα for Koga bases were linear within the range of 10 °C–40 °C when a mixture of equal volumes of methanol and 2-propanol was used as mobile phase on an amylose tris-(3,5-dimethylphenylcarbamate) stationary phase. Van’t Hoff plots became non-linear when 2-propanol concentration was increased to 85% while maintaining the other conditions unchanged. However, the plots could be divided into two linear regions, indicating that van’t Hoff plots are linear in a limited temperature range. The plots turned from one linear region to the other at 20 °C, which indicate a that change of conformation of AD phase and an alteration of the enantioseparation mechanism. Thermodynamic data obtained in this study provided additional understanding of enantioseparation of Koga bases through enthalphy and entropy considerations. Temperature effect on enantioseparation could be explained in great detail if conformational state of CSP, composition of mobile phase, and chromatographic characteristics of chiral analyte are known.
